# Versican Associates with Tumor Immune Phenotype and Limits T-cell Trafficking via Chondroitin Sulfate

**DOI:** 10.1158/2767-9764.CRC-23-0548

**Published:** 2024-04-03

**Authors:** Priyanka Hirani, Jacqueline McDermott, Vinothini Rajeeve, Pedro R. Cutillas, J. Louise Jones, Daniel J. Pennington, Thomas N. Wight, Salvatore Santamaria, Kimberly M. Alonge, Oliver M.T. Pearce

**Affiliations:** 1 Barts Cancer Institute, John Vane Science Centre, Queen Mary University of London, London, United Kingdom.; 2 Department of Histopathology, Imperial College Healthcare NHS Trust, London, United Kingdom.; 3 Centre for Immunobiology, Blizard Institute, Barts and the London Medical School, Queen Mary University of London, London, United Kingdom.; 4 Matrix Biology Program, Benaroya Research Institute at Virginia Mason, Seattle, Washington.; 5 Department of Biochemical Sciences, School of Biosciences, Faculty of Health and Medical Sciences, Edward Jenner Building, University of Surrey, Surrey, United Kingdom.; 6 Department of Medicinal Chemistry, University of Washington, Seattle, Washington.

## Abstract

Immunotherapies for cancers of epithelial origin have limited efficacy, and a growing body of evidence links the composition of extracellular matrix (ECM) with the likelihood of a favorable response to treatment. The ECM may be considered an immunologic barrier, restricting the localization of cytotoxic immune cells to stromal areas and inhibiting their contact with tumor cells. Identifying ECM components of this immunologic barrier could provide targets that whether degraded *in situ* may support antitumor immunity and improve immunotherapy response. Using a library of primary triple-negative breast cancer tissues, we correlated CD8^+^ T-cell tumor contact with ECM composition and identified a proteoglycan, versican (VCAN), as a putative member of the immunologic barrier. Our analysis reveals that CD8^+^ T-cell contact with tumor associates with the location of VCAN expression, the specific glycovariant of VCAN [defined through the pattern of posttranslational attachments of glycosaminoglycans (GAG)], and the cell types that produce the variant. In functional studies, the isomers of chondroitin sulfate presented on VCAN have opposing roles being either supportive or inhibiting of T-cell trafficking, and removal of the GAGs ameliorates these effects on T-cell trafficking. Overall, we conclude that VCAN can either support or inhibit T-cell trafficking within the tumor microenvironment depending on the pattern of GAGs present, and that VCAN is a major component of the ECM immunologic barrier that defines the type of response to immunotherapy.

**Significance:**

The response to immunotherapy has been poor toward solid tumors despite immune cells infiltrating into the tumor. The ECM has been associated with impacting T-cell infiltration toward the tumor and in this article we have identified VCAN and its structural modification, chondroitin sulfate as having a key role in T-cell invasion.

## Introduction

Across the subtypes of breast cancer, triple-negative (TNBC) is considered the most likely to be responsive to immunotherapy because of the higher level of immune infiltrate (1). Unfortunately, this has not resulted in dramatic clinical benefit in immunotherapy trials (2), likely because of the localization of immune cells, in particular cytotoxic T cells, which when infiltrated within the tumor microenvironment appear to be unable to make contact with tumor cells and therefore incapable of eliminating them (3). This spatial orientation described as immune-excluded likely explains why immunotherapy trials that stratify patients based solely on target markers (such as PDL1), have led to these powerful treatments being withdrawn. Within TNBC's, the immune-excluded phenotype is the most common of three broad tumor immune phenotypes (TIP), which summarize the location of immune cells with respect to the tumor epithelium. Meanwhile, tissues with the “inflamed” phenotype, which is the least common, exhibit a high level of immune cells spread throughout the tumor epithelium and stroma (4–6) and unlike the excluded phenotype are much more likely to exhibit a positive response to immunotherapy (7). The final phenotype is “desert” which shows an overall poor level of immune infiltrate in all regions that is thought to be due to a poor priming of adaptive immune response against the tumor, therefore leading to a poor response to immunotherapy (8, 9).

Understanding mechanisms underlying immune cell trapping within the stroma in excluded tissues could lead to potential targeted therapies to improve immunotherapy response. A major role may be exerted by the composition of the tumor extracellular matrix (ECM) which in excluded tumors behaves like an immunologic barrier restricting immune cell contact with tumor cells (10, 11) either through the effect on phenotype or migration of immune cells (12–14). Examples of how tumor ECM may influence immune infiltration include the differentiation of immune cells into tumor-associated phenotypes (15–17). The trafficking of immune cells may be influenced through physical barriers resulting from the organization of matrix fibers (18, 19) or by the manipulation of biochemical gradients that guide them toward the tumor (20, 21). The ECM molecules associated with these roles could therefore be potential therapeutic targets with the goal of generating tumor-suppressive immune phenotypes or breaking down barriers to immune cell trafficking to tumor cells.

In our previous work, we identified a molecular signature, present at both gene and protein level, describing a composition of tumor ECM associated with immune suppression and poor prognosis (14). Here we explore whether these molecules, including previously identified ECM targets fibronectin (FN1), cathepsin B (CTSB), and versican (VCAN), are associated with the TIPs. Using histopathologic analysis, TIPs were assigned to a library of TNBC tissues where the immune-excluded phenotype was identified as most common. Comparing TIP phenotype and the spatial arrangement of the selected ECM molecules we identified the chondroitin sulfate proteoglycan (CSPG) VCAN to be associated with T-cell infiltration and the immune-excluded phenotype. Moreover, we report here that tumor-expressed VCAN exhibits heterogenous structures through the combination of protein isoforms and attached CS-glycosaminoglycan (CS-GAG) sulfation patterning (22) which associates with T-cell infiltration in both tissue analysis and *in vitro* models. These data lead us to hypothesize that upregulation in VCAN expression may be associated with pathogenic CD8^+^ T-cell exclusion, and that specific alterations in CS-GAG chains could be targeted to improve T-cell migration into the tumor. To test this hypothesis, we determined changes in both the abundance of VCAN core protein isoforms and attached CS-GAGs in a subset of human TNBC tissues, and then associated these changes with the three TIP subtypes. Finally, we evaluated the changes in CS-GAG sulfation patterning specific to each TIP and evaluated the effect of differential CS-GAG on T-cell migration.

## Materials and Methods

### IHC

A total of 4-µm-thick human TNBC formalin-fixed paraffin-embedded (FFPE) tissue sections were obtained from the Breast Cancer Now Tissue Bank. Slides were deparaffinized with xylene and rehydrated through a descending ethanol series. Antigen retrieval was completed using a citrate-based unmasking buffer (pH6, H-3300, Vector) or Tris-EDTA–based buffer (pH9, ab93684, Abcam) in an antigen retriever (Aptum biologics) at 102°C for 15 minutes. Endogenous peroxidases were blocked with 0.3% hydrogen peroxide in methanol for 30 minutes. Endogenous phosphatases were blocked with dual endogenous block for 10 minutes (S2003, Agilent). A total of 2.5% goat serum was used to block tissues for horseradish peroxidase (HRP) staining and 2.5% horse serum buffer was used for alkaline phosphatase (AP) stains. Antibodies were diluted in blocking solution and incubated for 1 hour at room temperature or 4°C overnight. HRP stains were detected using Impress-HRP anti-rabbit (MP-7451, Vector). Conditions for each antibody are shown in Table 1. 3,3′-Diaminobenzidine reagent (DAB, K3468, DAKO) was used to detect the stain. For AP stains, Impress-AP anti-mouse/rabbit (MP5402, MP-5401, Vector) was used followed by detection with Vector Red (SK-5100, Vector). For dual staining, the HRP was detected prior to AP. Hematoxylin counterstain was completed followed by dehydration with an ascending ethanol series and clearing in Xylene. Slides were mounted and scanned using a Pannoramic scanner (3D Histech).

**TABLE 1:** Antibodies for IHC. Optimized parameters for each antibody

Marker	Antigen retrieval	Secondary	Dilution	RT or 4°C	Company	Catalog number	RRID
VCAN	pH6	Rabbit	1:500	RT	Sigma	HPA004726	AB_1080561
PanCK	pH6	Rabbit	1:1,000	RT	DAKO	Z0622	AB_2650434
FAP	pH6	Rabbit	1:250	4°C	Abcam	AB207178	AB_2864720
α-SMA	pH6	Mouse	1:2,000	RT	Sigma	A5228	AB_262054
CS	pH6	Mouse	1:600	RT	Abcam	AB11570	AB_298176
COMP	pH6	Rat	1:75	RT	Abcam	AB11056	AB_297708
FN1	—	Rabbit	1:500	RT	Sigma	F3648	AB_476976
CTSB	pH6	Rabbit	1:200	RT	Abcam	AB125067	AB_10972167
COL11A1	pH6	Rabbit	1:100	RT	Sigma	HPA052246	N/A
CD8	pH9	Mouse	1:500	4°C	DAKO	M7103	AB_2075537
CD68	pH9	Mouse	1:12,000	4°C	Thermo Fisher Scientific	14-0688-82	AB_11151139
BGN	pH6	Goat	1:500	RT	R&D	AF2667	AB_2065204
DCN	pH6	Rabbit	1:500	RT	Proteintech	14667-1-AP	AB_2090265
VCAN (DPEEAE)	pH6	Rabbit	1:400	RT	Abcam	AB19345	AB_444865

Abbreviation: RT, room temperature.

### RNAscope

ISH was completed following the manufacturers protocol (ACD Bio). Tissues were deparaffinized by incubating at 60°C for 1 hour followed by agitation in xylene for 5 minutes twice. Slides were rehydrated in 100% ethanol and left to air dry. RNAscope hydrogen peroxide was used to block background peroxidase. Target retrieval was completed at 95°C–100°C for 15 minutes. Slides were dipped in ethanol and left to air dry. Protease plus solution was added to slides and they were incubated in a HybEZ oven for 30 minutes at 40°C. Slides were washed and the VCAN probe was added to slides. Slides were incubated in the HybEZ oven for 2 hours at 40°C. Slides were washed and incubated overnight in 5X SSC solution (0.75 mol/L NaCl and 75 mmol/L trisodium citrate, pH 7) at room temperature. Sequential amplifiers were then added to amplify the hybridization signals. Slides were treated with AMP reagents at 40°C or room temperature for either 15 or 30 minutes as detailed in the manufacturer's protocol. The amplified signal was detected using DAB reagent. For dual RNAscope, the Red and Green detection reagents were used to detect the signals. Tissues were counterstained with 50% hematoxylin for 2 minutes at room temperature, followed by submerging slides in 0.02% ammonium. Slides were dehydrated in 70%, 90%, and 100% ethanol and cleared with xylene. Slides were mounted with DPX and scanned with a panoramic scanner.

### Cell and Matrix Quantification

The automated trained software QuPath (23) was used to calculate the number of immune cells and area of staining. Classification of tumor and stroma was guided by pancytokeratin (PanCK) stain. For the initial analysis to study the tumor composition, 4 mm^2^ squares were selected to be studied in the same areas across all stains. For the immune phenotype analysis, we looked to study the immune cells in the tumor islands and the stroma adjacent to the tumor epithelial cells. To achieve this, areas with 100 µm wide margins were drawn around the tumor margin to form the inner and outer invasive areas. Areas adjacent to this were then drawn around to form the tumor core and stroma, respectively. The area analyzed for the stroma and tumor core was up to 300 µm wide. For the analysis, the inner invasive and tumor core were averaged to form the epithelial zone and the outer invasive and stroma were averaged to form the stromal zone. The same areas were chosen to study CD8 and CD68 localization. Positive cell detection was used to count the number of CD8^+^ T cells and CD68^+^ monocytes while positive pixel detection was used to detect the percentage of VCAN as well as the tumor and stroma area. Multiple areas were surveyed within each tissue to account for Intratumoral hetereogeneity. The areas were averaged to obtain the overall TIP of the tissue with the phenotypes also determined for individual areas.

Colocalization analysis was completed by deconvolution of the stains on FIJI and overlaying the two stains. Color counter plugin was used to count the areas of staining for a single marker and colocalization.

### Gene Dataset Analysis

The Cancer Genome Atlas Breast Invasive Carcinoma Collection (TCGA-BRCA) datasets were aquired. TNBC cases were selected on the basis of the analysis from Lehmann and colleagues (24). A total of 25 samples were selected from each TNBC subtype identified. Single cell portal was used to plot data from Wu and colleagues looking at single cells from TNBC (25).

### Cell Culture

HCC38 cells (ATCC, RRID:CVCL_1267) were cultured in RPMI1640 medium supplemented with 1% penicillin-streptomycin, 1% l-glutamine, and 10% FBS (Gibco). MDAMB468 (ATCC, RRID:CVCL_0419) were cultured in DMEM supplemented with 1% penicillin-streptomycin, 1% l-glutamine, and 10% FBS. Mammary fibroblasts HMF3S (Applied Biological Materials) cultured in DMEM/F12 medium supplemented with 1% penicillin, 1% l-glutamine, and 10% FBS. Primary fibroblasts were obtained from the Breast Cancer Now Tissue Bank. Cells were grown in DMEM/F12 medium supplemented with 1% penicillin-streptomycin, 1% l-glutamine, and 10% FBS. Cells were routinely tested for *Mycoplasma* and authenticated though short tandem repeat sequencing.

### qRT-PCR

Cells were grown for 7 days in 6- or 24-well plates at 80% confluency. RNA was extracted from using the RNeasy Mini Kit (Qiagen), following the manufacturer's protocol. RNA concentration was determined using a Nanodrop 2000 spectrophotometer. With frozen tissues, samples were homogenized with a gentleMACs dissociator (130-093-236, Miltenyi Biotec) and Qiashredder (Qiagen) prior to RNA extraction. cDNA was generated from 500 ng to 1 µg RNA using the high-capacity cDNA reverse transcription kit (4368814, Thermo Fisher Scientific) using manufacturer's protocol. Samples were diluted with DNase/RNase-free water to give a concentration of 5 ng/µL cDNA.

Primers for VCAN isoforms were taken from previous publications (26, 27) and confirmed using NCBI blast for specificity, melting points, GC%, dimerization, and amplicon size (70–150 bp). Primers used in analysis shown in Table 2. SYBR green detection was used with 200 nmol/L forward and reverse primer. qRT-PCR on fibroblasts was completed on the StepOnePlus Real-Time PCR system (Applied Biosystems) with a holding stage (10 minutes at 95°C) and 40 cycles (15 seconds at 95°C and 1 minute at 60°C). Tissue RNA was run on a 384-well plate with a holding stage (10 minutes at 95°C) and 40 cycles (1 second at 95°C and 20 seconds at 60°C).

**TABLE 2:** Primers for qRT-PCR. Primer sequences for VCAN isoforms and housekeeping gene RPS13

Gene	Forward Primer	Reverse Primer	Amplicon bp
RPS13	TCGGCTTTACCCTATCGACGCAG	ACGTACTTGTGCAACACCATGTGA	153
V0	GCACAAAATTTCACCCTGACAT	TTAGATTCTGAATCTATTGGATGACCA	112
V1	CCCAGTGTGGAGGTGGTCTAC	CGCTCAAATCACTCATTCGACGTT	126
V2	CCCAGCAAGCACAAAATTTCAC	TAGGATAACAGGTGCCTCCGTT	122
V3	CCCTCCCCCTGATAGCAGAT	GGCACGGGGTTCATTTTGC	72
V4	CAGTACCACTGTTGAGGAAAAGAAAA	CGTTAAGGCACGGGTTCATT	86

### Versican Enrichment

Cells were grown in 15 cm cell culture dishes. After 2 days, the media was changed to protein-free media (Lonza, 12-727F) with 50 µg/mL ascorbic acid. The media was collected after 7 days and replaced. The media was then collected again after 7 days. Protease inhibitor (78429, Thermo Fisher Scientific) and Ethylenediaminetetraacetic acid (EDTA) was added to the media. Macro-prep DEAE beads (Bio-Rad) were washed with dH_2_O and wash buffer (0.15 mol/L NaCl, 0.05 mol/L Tris). Beads were mixed with the media for 3 hours at 4°C. The bead slurry was then poured into a gravity flow column. The flow through was collected and the beads were washed with wash buffer. The beads were then eluted with increasing salt concentrations (0.3–1.5 mol/L, 4 mol/L GuHCl) with 0.05 mol/L Tris. The elution fractions were tested for VCAN using a dot blot where 15 µL of each elution was pipetted onto an activated polyvinylidene difluoride membrane. The membrane was activated with methanol and washed with dH_2_O. Once the samples had diffused into the membrane, ponceau staining was completed and the membrane was probed for VCAN (12C5, DSHB, 1:500). The membrane was washed in water to remove the ponceau and blocked with 5% skimmed milk for 30 minutes. The primary antibody was added for 40 minutes at room temperature. The membrane was washed twice in TBST and then incubated with the secondary antibody for 30 minutes at room temperature. Chemiluminescence solution was used to detect protein with Amersham Imager 2100 (Supplementary Fig. S1A and S1B).

### Protein Mass Spectrometry

Samples were treated with 0.025 mol/L dithiothreitol, and the sample was incubated for 1 hour at 23°C in the dark. A total of 2 mmol/L iodacetamide was added with a similar incubation. Samples were then treated with 1 µg endoproteinase GluC (ProMega) for 16 hours at 37°C in the dark. A total of 20 mmol/L HEPES (-4-(2-hydroxyethyl)-1-piperazineethanesulfonic acid) buffer was used to dilute the sample 1:2. A total of 1,500 U of PNGase F (New England Biolabs) was added and the sample was incubated for 2 hours at 37°C in the dark. Chondroitinase ABC (ChABC; Sigma C3667) treatment was then completed with 0.01 U/mL ChABC in 40 mmol/L Tris-HCl pH8 and 40 mmol/L sodium acetate overnight at 37°C. The samples were then desalted with a C18 spin column and precipitated using a SpeedVac. Samples were reconstituted in 0.1% trifluoroacetic acid to give 1 µg/µL and 1 µL was run on the LC/MS-MS (Q-Exactive Plus connected to an Ultimate 3000 RSL nano LC, Thermo Fisher Scientific). Gradient elution was from 3% to 28% solvent B in 120 minutes at a flow rate 250 nL/minute with solvent A being used to balance the mobile phase (buffer A was 0.1% formic acid in water and B was 0.1% formic acid in acetonitrile). The spray voltage was 1.95 kV and the capillary temperature was set to 255°C. The Q-Exactive plus was operated in data-dependent mode with one survey mass spectrometry (MS) scan followed by 15 MS-MS scans. The full scans were acquired in the mass analyzer at 375–1,500 m/z with the resolution of 70,000, and the MS-MS scans were obtained with a resolution of 17,500.

MS raw files were converted into Mascot Generic Format using Mascot Distiller (version 2.8.1) and searched against the SwissProt database (SwissProt_2021_02. fasta) restricted to human entries using the Mascot search daemon (version 2.8.0). Allowed mass windows were 10 ppm and 25 mmu for parent and fragment mass to charge values, respectively.

### CS-GAG MS

#### FFPE tissues

Two, 4 µm FFPE tissue sections were taken for each sample. PanCK staining was completed on a consecutive slide to identify tumor versus stroma zones within the tissues. Sections were first deparaffinized and rehydrated before washing thrice for 10 minutes in MS-grade H_2_O and then 10 minutes in 50 mmol/L ammonium bicarbonate buffer (pH 7.6). Using a hydrophobic pen, margins were drawn around tumor zones. ChABC was dissolved in 50 mmol/L ammonium bicarbonate buffer to make a final concentration of 0.5 U/mL and then added to the outlined tumor regions and left at 37°C overnight. The digested CS-GAG disaccharides were collected, tissue washed for 10 minutes in 50 mmol/L ammonium bicarbonate buffer, and ChABC was added a second time to the remaining, nondigested stroma regions and incubated at 37°C overnight. The second set of stroma-digested CS-GAG disaccharides were collected and then freeze-dried. The volume was brought up to 30 µL with MS-grade H_2_O (Supplementary Fig. S2).

#### Cell Culture

Media was collected from cell cultures and free-floating protein were concentrated and dialyzed using a 10K centrifugal column. The samples were dialyzed 3x against MS-grade H_2_O and 1x ammonium bicarbonate buffer. 0.5 U/mL ChABC was added to the dialyzed sample to bring up the volume to a total of 500 µL and incubated overnight at 37°C. After digesting the media CS-GAGs, the CS disaccharide units were collected using a 3 KDa centrifugal column at 14,000 × *g* for 10 minutes. The samples were freeze dried and brought up to 30 µL with MS-grade H_2_O as described above.

The samples were run on LC/MS-MS as described in Alonge and colleagues (28).

### T-cell Isolation and Activation

Peripheral blood mononuclear cells were isolated from leukocyte cones derived from healthy patients. Pan-T cell negative selection was performed using EasySep Human T Cell Isolation Kit (Stemcell) according to manufacturer's protocol. T cells were then activated using the T Cell TransAct beads (Miltenyi Biotec) for 4 days. Flow cytometry was used to confirm purity of sample and activation of cells.

### Transwell Assay

A total of 5 µg of ChABC treated and untreated crude and enriched VCAN protein from HCC38 and HMF3S cell lines were taken and added to 0.05% collagen gel solutions. A total of 20 µL collagen gels were set onto wells of a chemotaxis plate (Sartorius, 4582). The gel was set for 1 hour and then 30 µL serum-free DMEM/F12 media was added on top. DMEM/F12 media with serum and 100 ng/mL SDF1 was added to the bottom well. T cells were then seeded on top of the gels at 10,000 cells per well. The plate was imaged via incucyte (Sartorius) for over 24 hours with images taken every 1.5–2 hours. The number of T cells migrated through the collagen gel were counted at each timepoint (Supplementary Fig. S3).

### Statistical Analysis

All the graphs and statistical tests were done on GraphPad Prism V9. All correlations were determined using Spearman rank correlation test. For comparing paired data among groups, Repeated measures one-way ANOVA was used. Unpaired data were calculated using one-way ANOVA or two-way ANOVA with either Tukey test or Welch correction depending on the group size and variations amongst samples. Data were statistically significant if the *P* value <0.05.

### Data Availability

RNA sequencing data were obtained from TCGA BRCA project. Patients with TNBC were identified from the analysis completed by Lehmann and colleagues (29). Single-cell RNA sequencing (scRNA-seq) data are available from the Single Cell Portal (25).

## Results

### The Excluded Immune Phenotype Makes Up the Majority of TNBC Cases Tested

**FIGURE 1:**
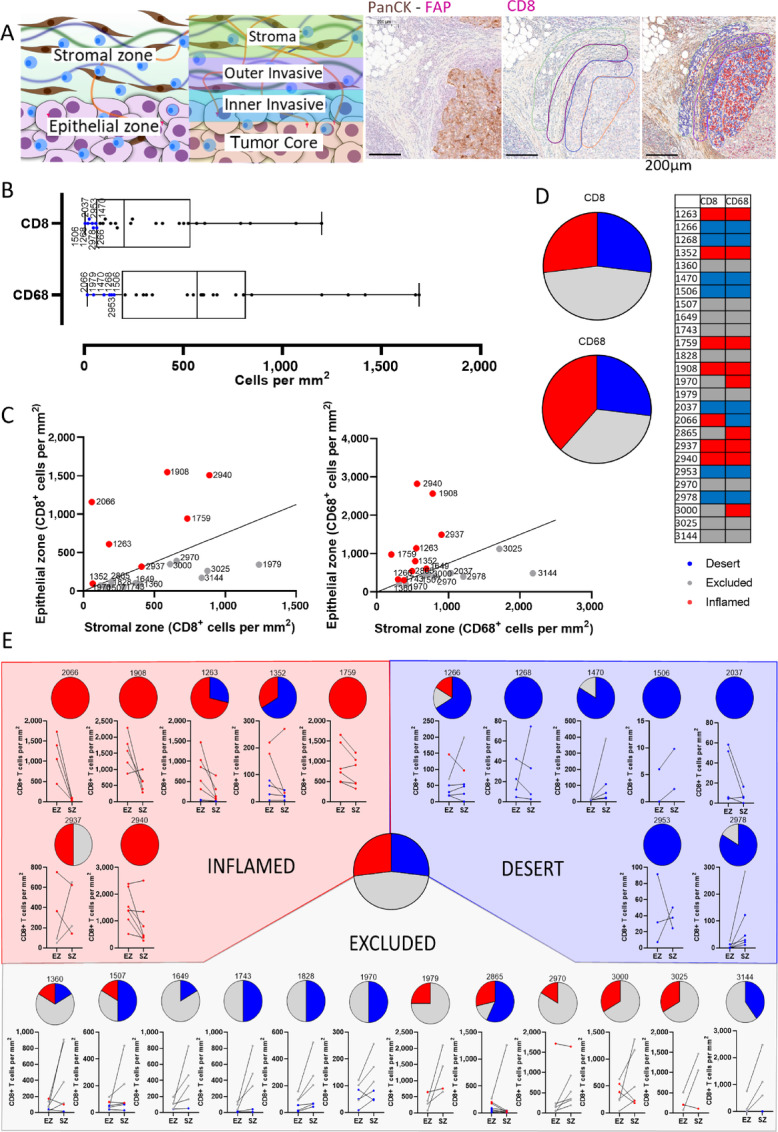
Determining the immune phenotype of tumor tissues. A total of 26 TNBC tissues were analyzed for the TIP. **A,** Annotation of slide on QuPath for analysis of the four regions: Stroma, Outer Invasive, Inner Invasive, and Tumor Core. Number of cells per mm^2^ and VCAN percentage were counted in these regions. **B,** Box plot showing desert tissues under the lower quartile. **C,** Comparison of cells in the stromal zone and epithelial zone to determine excluded and inflamed tissues. Line indicates a ratio of 0.75. **D,** Pie charts showing distribution of phenotypes for 26 tissues. Table showing phenotype per tissue for CD8^+^ T cells and CD68^+^ macrophages. **E,** Pie charts showing phenotypes within each tissue. Graphs comparing levels of CD8^+^ T cells in the epithelial zone (EZ) and stromal zone (SZ).

To explore how ECM components associate with immune cell infiltration, we first characterized the TIP of a library of 26 primary TNBC tissues. IHC established tumor (PanCK) and stromal regions (fibroblast activation protein, FAP) of the tumor, as well as cytotoxic T cell (CD8) and macrophage (CD68) infiltration (Fig. 1A). Cells were counted (cells/mm^2^) across four defined regions within the tissue including (i) tumor core, that is, areas where tumor epithelial cells are present, (ii) inner invasive and (iii) outer invasive, which describes the leading edge of the tumor epithelium, and (iv) stroma, which defines the region away from the tumor epithelium (Fig. 1A). The data from these regions were used to define each tissue under the three broad TIP phenotypes—inflamed, excluded, and desert. Initially, the analysis method from Kather and colleagues (5) was used to measure the TIPs; however, within our tissue library, comparisons of the total CD8^+^ T cells in each phenotype suggested that phenotypes may have been assigned on the basis of the number of cells as opposed to location (Supplementary Fig. S4A). To overcome this limitation, we adapted an analysis method that first identified the desert tumors based on cell number (i.e., below the lower quartile of the subset; Fig. 1B), and then computed a ratio of the number of immune cells in the epithelial zone (tumor core and inner invasive regions) and stromal zone (outer invasive and stroma regions). A ratio below 0.75 was used as a cutoff for excluded tissues (Fig. 1C). No significant difference was observed between the number of immune cells infiltrating in the excluded and inflamed tissues, and therefore the phenotypes could be defined on cell location alone (Supplementary Fig. S4B). The excluded phenotype made up the majority of the tissues at 46%, with inflamed and desert tumors equal at 27%. Repeating this same analysis for CD68^+^ macrophages showed an equal share of tissues were inflamed or excluded for this marker (35% each), with desert making up the smallest slice (27%). When comparing the TIPs between CD8 and CD68, 85% of tissues had comparable phenotypes indicating that features that exclude T cells may have a lesser effect on CD68^+^ cells (Fig. 1D). During the analysis we noted that the immune phenotype analysis varied across a tissue section, with 69% of tissues exhibiting two or more phenotypes. This heterogeneity occurred in all three immune phenotypes, and in particular all excluded tissues contained two or more phenotypes (Fig. 1E; Supplementary Fig. S4C). In some cases, a tissue may be assigned to a different TIP even though there are more sites detected with a different phenotype, for example, tissue 1352 (Fig. 1E). This is because the overall phenotype was determined using an average of the CD8^+^ T-cell counts and therefore a few areas with high counts would lead to the tissue being classed as inflamed despite having more desert areas.

To test whether the immune phenotypes are conserved throughout a patient's tumor, we repeated this analysis again either on a section of tissue taken within the same tissue block, or a new tissue block from the same patient. New sections from the same tissue block gave a consistent immune phenotype with 83% of tissues sharing the phenotype (Supplementary Fig. S4D), but sections from new tissue blocks only shared the phenotype in 57% of cases (Supplementary Fig. S4E). This indicates that TIPs are likely to be conserved over small distances, but across a larger area, tumors may become enriched for another immune phenotype. In summary, using spatial analysis we were able to classify TNBC tissues for their TIP and demonstrate how this phenotype can vary throughout a tissue. The heterogeneity in TIPs within a tissue section and across different pieces of tissues may explain why residual disease is reported in tumors after cell and antibody-based immunotherapy (30). Following TIP classification, we investigated what ECM molecules may be associated with CD8^+^ T-cell localization in tumors and how the molecule is expressed in the different TIPs.

### VCAN Associates with T-cell Localization

**FIGURE 2:**
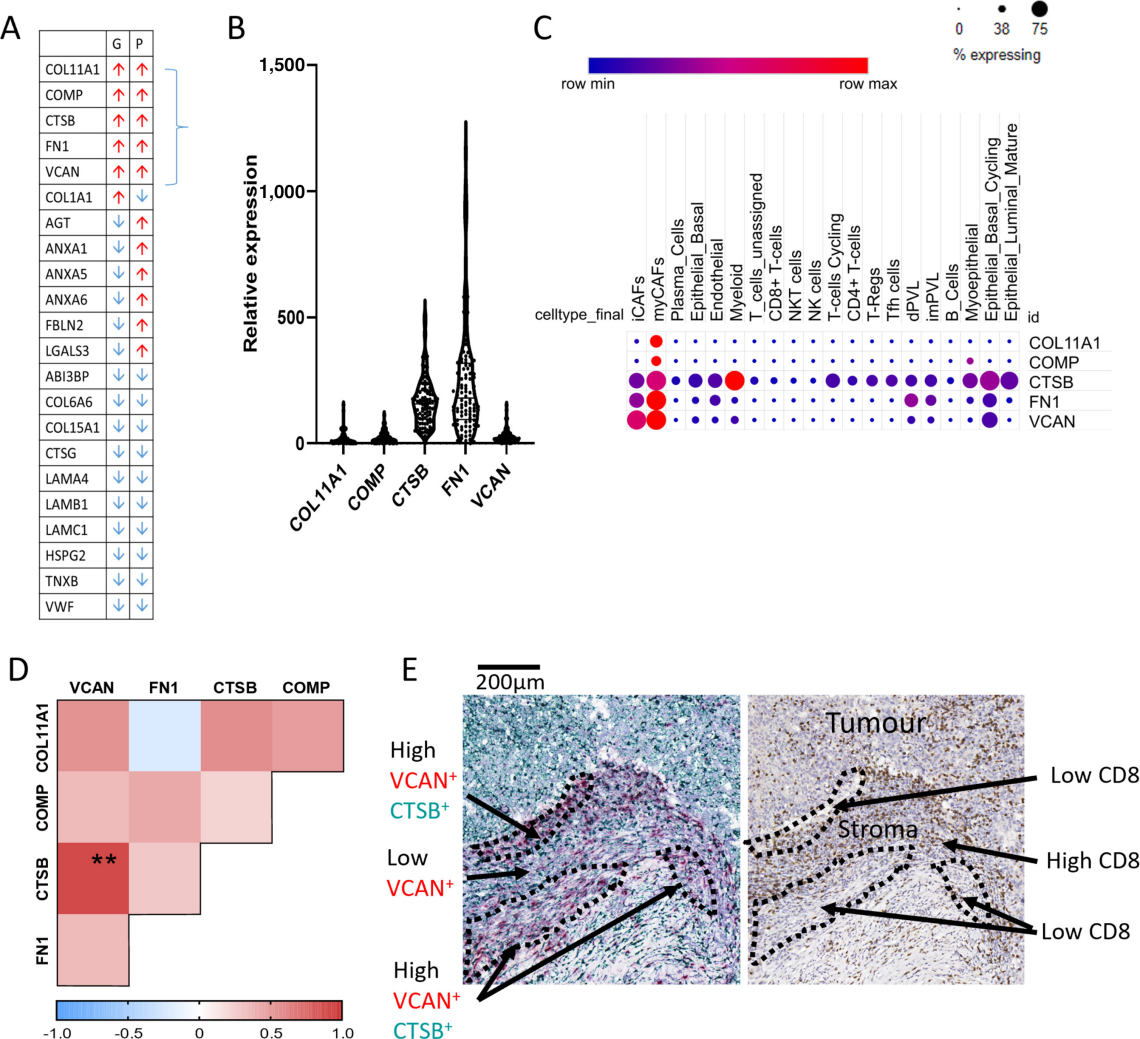
VCAN is associated with T-cell localization. **A,** Matrix index from Pearce and colleagues (14). Arrows indicate upregulation or downregulation at gene (G) or protein (P) level. **B,** Level of gene expression of top five upregulated molecules in 100 samples from TCGA dataset. **C,** scRNA-seq expression of the five genes indicating which cell types express the genes and the percentage of those cells that are positive. Data obtained from Wu and colleagues (25). **D,** IHC completed on consecutive TMA cores for COL11A1, COMP, CTSB, FN1, and VCAN. Spearman rank correlation of the expression of proteins from IHC analysis of eight TMA cores. **, *P* < 0.01 (*N* = 8). **E**, Comparison of dual RNAscope for CTSB (green) and VCAN (red) with IHC staining for CD8.

Previous work by our lab and others has identified matrix signatures that associate with immunosuppression and immunotherapy response (14, 31). We started with a previously defined ECM signature that also associates with poor prognosis across 12 epithelial cancers and was particularly strong in TNBC samples (Fig. 2A; ref. 14). We turned our attention to five molecules within the signature, COL11A1, COMP, CTSB, FN1, and VCAN, which were upregulated at both gene and protein level. We first analyzed the expression and cellular origin of these molecules using gene level analysis from TCGA datasets and publicly available scRNA-seq data (25). These data indicated FN1 and CTSB were the most highly expressed followed by VCAN, then COL11A1, and finally COMP (Fig. 2B). Single-cell data indicated that the main source of all five proteins was myofibroblasts (myCAF). Inflammatory fibroblasts (iCAF) also appeared to contribute to VCAN, CTSB, and FN1 expression. VCAN and FN1 also appeared to originate from epithelial cells, whilst CTSB was produced by the majority of the cell types analyzed, in particular myeloid cells (Fig. 2C).

Next, we compared the protein expression of these five proteins within a tissue microarray and analyzed their relative location with one another within the tissue (Supplementary Fig. S5A). Comparison of the abundance of each protein indicated a particularly strong correlation between VCAN and CTSB (Fig. 2D). The IHC stains for the five ECM proteins were overlaid to identify levels of colocalization. FN1 was presented in a uniform fashion across a tissue which led to high levels of colocalization with each protein. Interestingly, VCAN and CTSB were found to have similar levels of colocalization with the other proteins. Despite this, the level of colocalization between VCAN and CTSB was lower than expected and may be due to low levels of expression in some cores (Supplementary Fig. S5A and S5B). To then study whether the correlation of CTSB and VCAN is linked to coexpression, tissue sections were analyzed using dual RNAscope. As indicated from the single cell data (25), *CTSB* was seen to be expressed by most cell types. In contrast, *VCAN* expression was limited predominately to the stroma. We then questioned whether these ECM genes (e.g., *VCAN* and *CTSB*) associated with CD8^+^ T cells due to their negative association with cytotoxic T-cell signatures (14). A consecutive section stained for CD8 and showed tissue regions exhibiting high *VCAN* expression appeared to have low or no CD8^+^ T cells present (Supplementary Fig. S5C). In contrast, when VCAN-positive (VCAN^+^) cells were around the tumor border, we observed less CD8^+^ T cells present in the tumor parenchyma, thus suggesting VCAN expression on the tumor border may regionally restrict T cells to the stroma preventing access to tumor cells (Fig. 2E). These results indicated that VCAN may play a critical role in restricting T-cell access to the tumor parenchyma in immune-excluded tumors.

### VCAN Expression in the Epithelial Zone Correlates with an Excluded Phenotype

**FIGURE 3:**
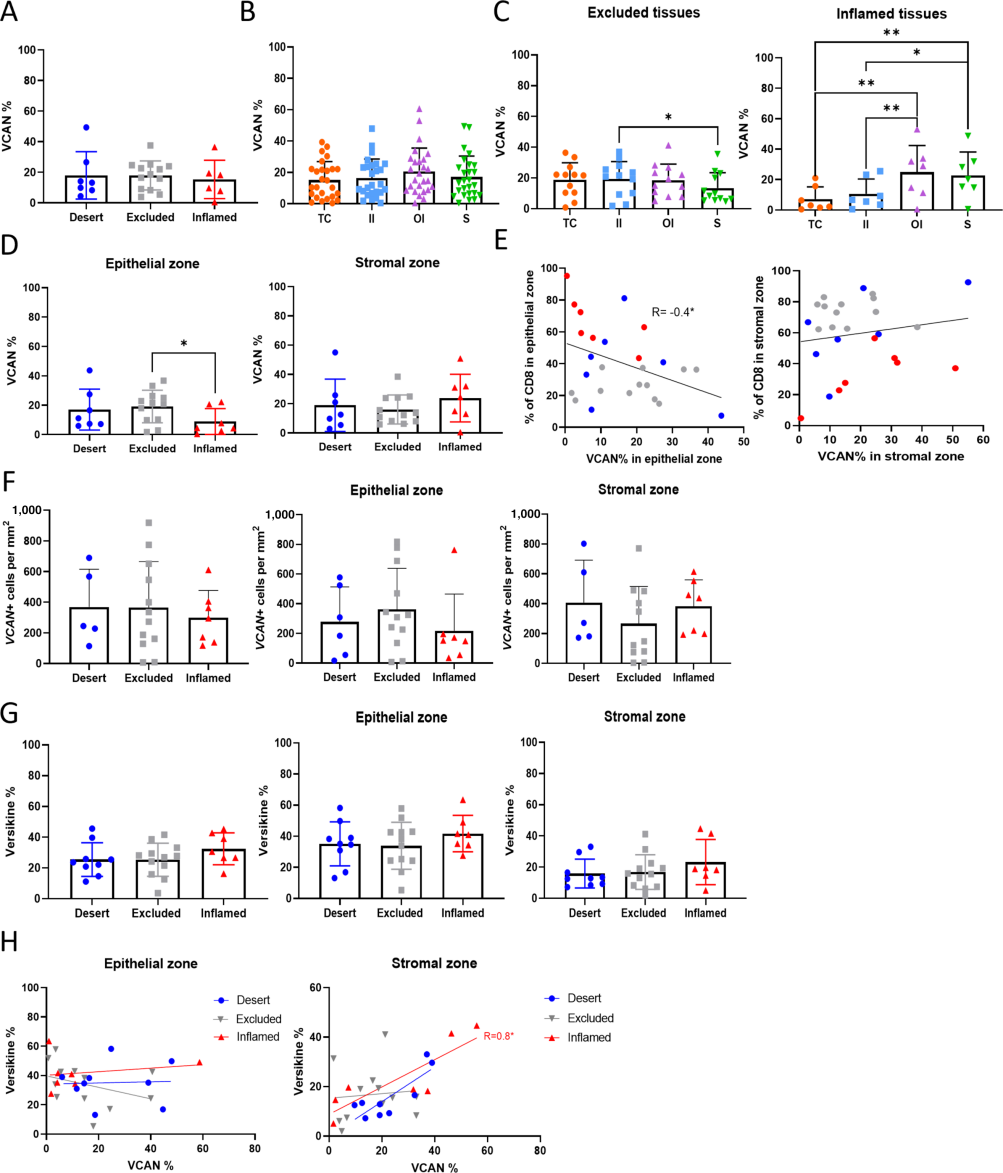
VCAN expression in different phenotypes and areas. IHC staining for VCAN completed on 26 tissues. Phenotypes for tissues based on CD8^+^ T-cell analysis. Images analyzed for VCAN percentage in whole area (**A**), each region (**B**), in areas split by phenotype (**C**) and in each phenotype split by zone (**D**). One-way ANOVA. **E,** Percentage of CD8^+^ T cells compared with percentage of VCAN in each zone. Phenotypes indicated by color of dot. Spearman rank correlation. **F,** Number of VCAN^+^ cells determined from RNAscope. Comparison between phenotypes in all areas and in specific zones. **G,** Versikine levels determined from IHC staining on 30 tissues. Comparison between phenotypes in all areas and in specific zones. **H,** VCAN stained on a consecutive slide. Comparison of VCAN and versikine in the different zones. Spearman rank correlation completed for each phenotype. *, *P* < 0.05; **, *P* < 0.01.

Having identified VCAN as a potential component of an excluded ECM, the levels of expression were examined in the different TIPs determined previously from CD8^+^ T-cell IHC (Fig. 1). We stained our library of 26 immune-phenotyped tissues for VCAN and found the overall VCAN expression level was similar across all three TIPs (Fig. 3A; Supplementary Fig. S6A). Next, we analyzed VCAN expression levels in the tissue regions outlined during the TIP analysis: tumor core, inner and outer invasive, and stroma (Fig. 1A). Overall, when analyzing all tissues together, there was no difference in VCAN expression levels across these regions (Fig. 3B). However, when we compared the regional expression of VCAN within each TIP, we noticed a trend in excluded tissues to exhibit higher VCAN expression within epithelial areas, and that this expression appeared to decrease toward the stroma (Fig. 3C). Conversely in inflamed tissues, there were low levels of VCAN expression within the tumor core which increased further away from the core (Fig. 3C). By comparing VCAN expression across TIP phenotypes, we found significantly less VCAN expressed within the epithelial zone of inflamed tissues compared with excluded and similar VCAN levels within the stromal zone across all TIPs (Fig. 3D; Supplementary Fig. S6B; and for images Supplementary Fig. S7). A significantly negative correlation was observed between the level of VCAN and percentage of CD8^+^ T cells in the epithelial zone but no correlation was found in the stromal zone (Fig. 3E; Supplementary Fig. 6C), further indicating that it is specifically high levels of VCAN in the epithelial zone that associate with altered immune infiltration. Next, we performed RNAscope and compared the numbers of VCAN^+^ cells by TIP on the same tissues. Here, we identified a trend in VCAN expression by RNAscope between the three phenotypes; where inflamed tissues tend to have less VCAN^+^ cells in the epithelial zone and excluded tissues less VCAN^+^ cells in the stromal zone (Fig. 3F), which fitted well with the protein level analysis. Finally, we analyzed the levels of the VCAN cleavage product versikine across TIPs and within the epithelial zone and stromal zone. Versikine has been previously shown to support a T-cell response against tumors (32) and we wondered whether inflamed tissues may exhibit higher levels of versikine. Our analysis showed that although versikine expression was not altered across TIP types (Fig. 3G), we observed a positive correlation between VCAN and versikine expression in the stromal zone only in inflamed tissues (Fig. 3H). As such, the high levels of the VCAN detected in the inflamed stroma might represent the degraded and T-cell supporting form. In summary, the spatial expression of VCAN within a tissue associates with TIP, excluded tissues displaying higher levels of VCAN in the epithelial zone and inflamed tissues predominantly expressing VCAN in the stroma. To understand how increased expression of VCAN in the epithelial zone may influence immune exclusion and whether the stromal VCAN has any impact we next analyzed VCAN isoform expression and glycosylation patterns in tissues and cell lines.

### VCAN Expression in Excluded Tissues is not Isoform-specific

**FIGURE 4:**
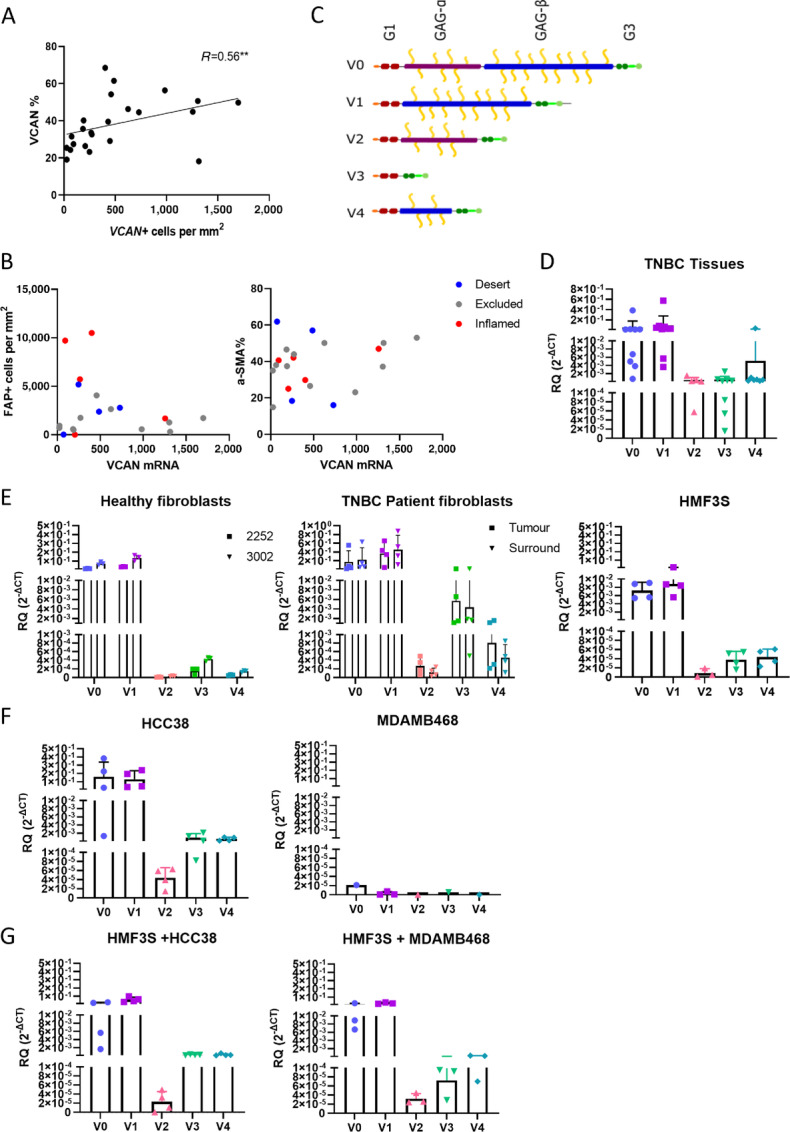
Expression of VCAN in tissues and cells. **A,** VCAN protein expression from IHC compared with gene expression from RNAscope. Spearman rank correlation. **B,** Comparison of FAP^+^ cells and percentage of α-SMA staining with number of VCAN^+^ cells. Each dot is colored to represent TIP. Red = inflamed, Gray = excluded, Blue = desert. **C,** Structure of VCAN isoforms and the number of predicted CS chains attached. qRT-PCR for VCAN isoforms completed on RNA extracted from frozen TNBC tissues (*N* = 9; **D**), fibroblasts (**E**), primary fibroblasts from healthy patients (*N* = 2) and from paired tumor and surround areas of patients with TNBC (*N* = 4), immortalized mammary fibroblast cell line (HMF3S; *N* = 4; TNBC cell lines HCC38 and MDAMB468 (*N* = 4; **F**). Coculture of HMF3S with HCC38 and MDAMB468 at a 4:1 ratio (*N* = 4; **G**). CT values for each gene normalized to CT values for housekeeping gene RPS13 to form ΔCT. **, *P* < 0.01.

scRNA-seq showed that VCAN is mostly expressed by CAFs and epithelial cells (Fig. 2C). We next used a combination of RNAScope (for VCAN) and IHC (PanCK, FAP, α-SMA, VCAN) to identify whether the TIP influences cellular VCAN expression. We first investigated the correlation between VCAN at both the gene and protein levels and found a significant correlation between genetic and protein expression within the tissue (Fig. 4A). Next, we tested whether the number of VCAN^+^ cells associated with two fibroblast markers (α-SMA and FAP) that have previously been shown to be markers for CAF populations in TNBC and associate with exclusion in TNBC. In both cases, no association was observed overall or when grouped by TIP phenotype (Fig. 4B). From the VCAN RNAscope staining in excluded tissues, we observed VCAN to be expressed around the tumor epithelial borders whilst in inflamed tissues VCAN^+^ cells were spread across the stroma (Supplementary Fig. S4).

VCAN isoforms are produced by exon splicing and differential translation of the GAG-α and GAG-β domains. The presence or absence of these two domains affects not only size of the VCAN core protein, but also the amount of attached CS-GAG chains (Fig. 4C; ref. 33). To explore VCAN isoforms in TNBC, qRT-PCR analysis was conducted on RNA extracted from 10 frozen primary TNBC tissues. V0 and V1 were identified as the highest expressed with the V2, V3, and V4 isoforms expressed in lower amounts and in some tissues were not expressed (Fig. 4D). Next, we compared these data against VCAN isoforms produced from fibroblasts (primary isolated from healthy tissues, paired tumor and surround tissue, and a human mammary fibroblast line, HMF3S), or TNBC cell lines (HCC38 and MDAMB468). The pattern of expression was similar across different cell lines, with the exception of MDAMB468 which produced only very low levels of all VCAN isoforms. Comparing the pattern of expression of these VCAN isoforms in fibroblasts (Fig. 4E) and tumor (HCC38; Fig. 4F) monocultures showed similar V0, V1, V3, and V4 expression as observed in tissues, but lower V2 expression. Cocultures of HMF3S and TNBC cell lines were analyzed to understand whether the cross-talk between the cells can affect the expression of specific isoforms. In the cocultures, increased VCAN isoform expression was observed when compared with HMF3S cells alone. Surprisingly, a cell line–specific effect was not observed and HMF3S cells were stimulated to produce similar levels of VCAN with each cell line (Fig. 4G). Overall, we concluded that the pattern of VCAN isoforms produced in the tissue were not associating with particular TIPs, and tumor–fibroblast interactions were also not driving a specific pattern of isoforms or altering VCAN expression.

### VCAN is the Predominant CSPG Associated with TIPs

**FIGURE 5:**
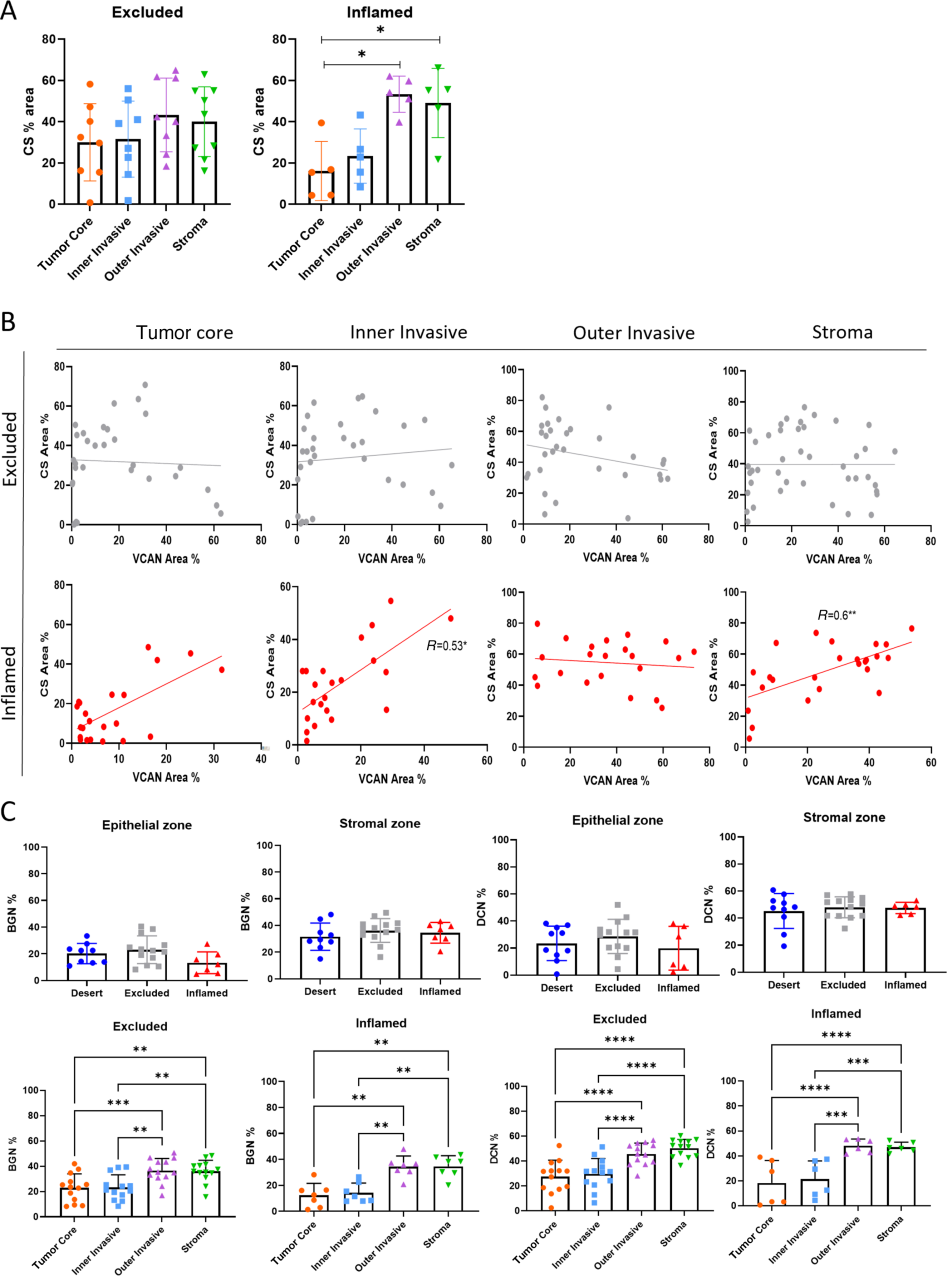
VCAN, CS, and CS-proteoglycans in different phenotypes. IHC completed for VCAN and CS on consecutive sections of 26 TNBC tissues. **A,** Spread of CS levels across regions in excluded and inflamed tissues. Tissues were analyzed by IHC using the CS56 antibody which can detect both A and C isomers of CS. **B,** CS and VCAN percentage determined from the same areas of consecutive slides. Correlations determined for stains in each region of excluded and inflamed tissues. Spearman rank correlation. **C,** IHC staining completed for BGN and DCN on 30 TNBC tissues. Percentage of stains compared between phenotypes and regions. RM one-way ANOVA. *, *P* < 0.05; **, *P* < 0.01; ***, *P* < 0.005; ****, *P* < 0.001.

With no potential associations observed with specific isoforms and TIP, we turned our attention to the CS-GAG glycosylation pattern of VCAN. The VCAN core protein is decorated with large polymers of CS-GAGs. These CS-GAG chains are attached to serine residues in their GAG-α and GAG-β domains. There are up to 22 predicted sites for CS glycosylation on VCAN (22). Using IHC, we compared CS across the four tissue regions for both excluded and inflamed tissues across 26 TNBC tissues. Here we observed CS levels to be consistent across the four regions in excluded tissues while in inflamed tissues there was a significant increase of CS toward the stroma (Fig. 5A). In excluded tissues, we observed no correlation between VCAN and CS-GAGs; however, in inflamed tissues there was a positive correlation between VCAN and CS-GAGs in the stroma and inner invasive region (Fig. 5B). The lack of correlation between VCAN and CS-GAGs in the excluded tissues suggests that other CSPG proteins that express CS-GAGs may be present in those tissues. To identify additional CSPGs expressed within TNBC, protein samples from cell line HCC38 were enriched for VCAN and other CSPGs using ion exchange chromatography (Supplementary Fig. S1A and S1B). MS then identified additional CSPG proteins within the sample including the small leucine-rich proteoglycans biglycan (BGN) and decorin (DCN; Supplementary Fig. S1C). Following this analysis, we then compared the expression of BGN and DCN across TIPs. Unlike VCAN, there was no specific pattern for expression of BGN and DCN in the different TIPs as they were significantly higher in the stroma for both excluded and inflamed phenotypes (Fig. 5C), indicating that VCAN is the primary CSPG that associates with TIP. We now predict that the lack of correlations between VCAN and CS-GAGs in the excluded tissues may be due to differences in CS-GAG length or number of chain attachment. Overall, these data indicate an association between CS expressed on VCAN and TIP phenotype.

### CS-A/C Ratio is Associated with Immune Exclusion and Poor T-cell Invasion

**FIGURE 6:**
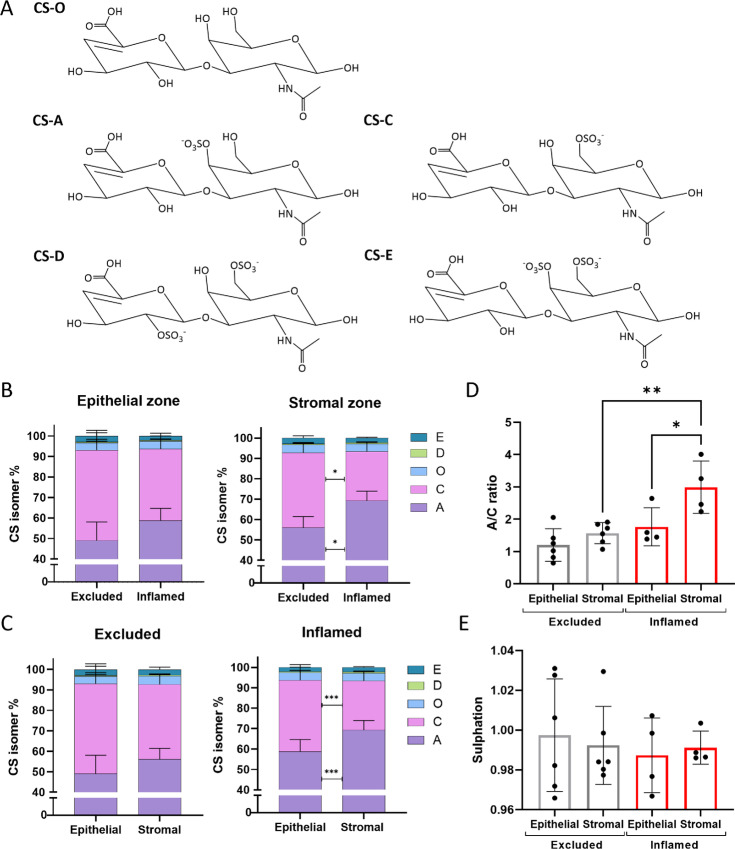
CS isomers expression in tissues. **A,** Structure of CS isoforms following digestion of the chains with ChABC. CS extracted from FFPE tissues in the epithelial zone and stromal zone. CS isoforms analyzed by MS to identify proportion of each isomer. **B,** Comparison of isoforms in excluded and inflamed tissues within the different zones. Two-way ANOVA. **C,** Comparison of isomer in the different zones within excluded and inflamed tissues. Two-way ANOVA. **D,** CS-A/C ratio determined for the different regions of each phenotype and compared. ANOVA. **E,** Level of sulfation per CS disaccharide in the different regions of each phenotype compared. *, *P* < 0.05; **, *P* < 0.01; ****, *P* < 0.001.

We next investigated whether VCAN-attached CS-GAGs exhibited intraregional sulfation pattern differences throughout the tumor. The biological function of VCAN-containing ECMs are dependent on the incorporation of differentially sulfated CS units into the attached polymers, including nonsulfated [CS-O (0S)], monosulfated [CS-A (4S) and CS-C (6S)], and disulfated [CS-D (2S6S) and CS-E (4S6S)] isomers (ref. 34; Fig. 6A). We hypothesized that differential CS-GAG sulfation patterning may, (i) associate with TIPs and (ii) exhibit intraregional differences throughout the tissue. To differentiate between tissue subregions, CS disaccharides were extracted from tumor and stromal areas of the tissue (Supplementary Fig. S2). MS quantification of the relative abundance of CS isomers shows that the monosulfated CS-A and CS-C isomers represented the major CS units in both epithelial and stromal zones of the tissue, with <10% comprised of CS-O, CS-D, and CS-E. Whilst we observed no difference in CS-GAG sulfation patterning in the epithelial zones between excluded and inflamed tissues, we report a significance decrease in CS-A and corresponding increase in CS-C in the excluded tissues within the stromal zone (Fig. 6B). Closer analysis shows a shift in CS isomers to favor the monosulfated CS-A in the stromal zone that was specific to inflamed TIP tissues (Fig. 6C). The change in the presence of the CS-A and C isomers between excluded and inflamed tissue was approximately 15% for each isomer. Inflamed tissues had a significantly higher ratio of CS-A/CS-C within the stromal zone compared with its epithelial zone, as well as the stromal zone of excluded tissues (Fig. 6D). The shift from monosulfated CS-C to CS-A isomer units did not result in a significant difference in overall CS-GAG sulfation levels (Fig. 6E), indicating CS-GAG anionic charge remains the same between tissue subregions and TIP. Overall, results from the analysis show that inflamed stromal subregions exhibit increased VCAN expression (Fig. 3C) in association with altered CS-GAG sulfation patterning (Fig. 6), which may impact immune cell migration into the tumor core.

**FIGURE 7:**
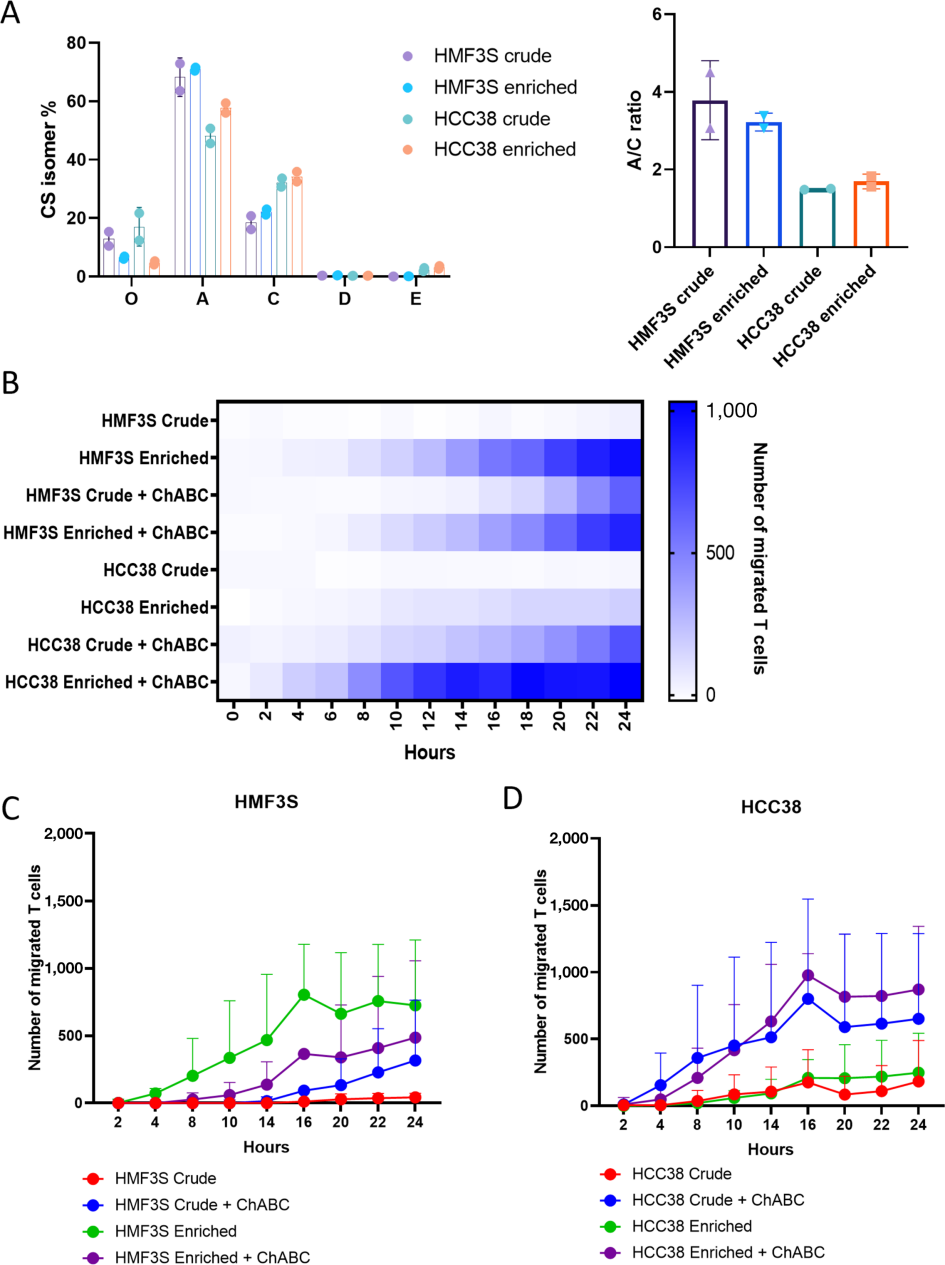
Effect of CS toward T-cell invasion. **A,** CS MS completed on the crude and enriched protein from HMF3S and HCC38 cell lines. CS isomers and A/C ratio determined. **B,** Transwell assay completed with 5 µg of each protein sample. Each sample was treated with ChABC for comparison. Heat map shows level of T cells invaded over time for one T-cell donor. Graphs show the level of migrated cells for HMF3S (**C**) and HCC38 (**D**) with error bars showing replicates with different T-cell donors.

As aforementioned, the biological function of VCAN-containing ECMs is dependent on the differentially sulfated CS isomers which have structural-dependent protein–glycan interactions. Resulting from these unique disaccharide isomer conformations, monosulfated CS-A and CS-C exhibit differential binding interactions to extracellular proteins, including increased CS-C binding and trapping of extracellular ligands such as chemokines and CD44 (35–37). One function of CS-C trapping of immune cell chemokines is to produce gradient CS-C “roadmaps” for immune cell migration such that immune cells migrate from lower to increasing concentrations of CS-C entrapped chemokines (Supplementary Fig. S8). Inspired by our previous results that showed the loss of this CS-C/chemokine roadmap in excluded TIP, we questioned whether this was due to trapping and mislocalization of the T cells in the CS-C enriched stroma, an effect circumvented by the intact CS-C gradient present in the inflamed tissues (Fig. 6). To test this possibility, we used a transwell assay where T-cell invasion through a collagen gel supplemented with protein enriched for VCAN from tumor cells (HCC38) and fibroblasts (HMF3S) over a 24-hour timeframe. HCC38 and HMF3S produce patterns of CS that are similar to those found in excluded versus inflamed tissues, that is HMF3S exhibit a higher A/C ratio as seen in inflamed tissues (Fig. 7A), compared with HCC38 which have a lower A/C ratio similar to excluded tissues. We found that gels supplemented with enriched VCAN presenting a low CS A/C ratio had a reduced level of T-cell invasion whereas enriched VCAN with a high CS A/C did support T-cell invasion. Next, we tested whether CS was directly involved in the support of T-cell invasion through the gel by enzymatically removing the CS-GAG chains from the enriched VCAN preps via ChABC digestion. Removal of high A/C CS chains from HMF3S matrix led to a slight reduction in T-cell migration through the gel indicating that this pattern of CS was supporting T-cell invasion (Fig. 7B and C). In contrast, removal of low A/C CS from HCC38 VCAN enriched matrix improved T-cell migration through the gel, indicating that this pattern of CS was inhibiting T-cell invasion (Fig. 7B and D).

## Discussion

In this study, we determined the role of VCAN in making the immunologic barrier that defines inflamed-excluded tumor phenotypes. We first determined the TIP of the tumors, building on previous methods presented in the literature (38). Early methods for this analysis were completed on colorectal cancer by Galon and colleagues (39), and in a pan-cancer study by Kather and colleagues (5), neither of which included TNBC and may explain why we found the method unsuitable for TNBC tissues. In particular, we found the previous reported method could not deduce whether the exclusion phenotype was due to stromal restriction or low immune infiltration. By using a ratio of immune cells in the epithelial zone and stromal zone to determine the excluded and inflamed tissues, this limitation could be overcome. From our subset, only a quarter of patients were found to have inflamed tumors which reflects the immunotherapy response rates observed in clinics for this indication (40). Other articles have recently looked at immune exclusion in TNBC, including Gruosso and colleagues (4) and Hammerl and colleagues (8). In Gruosso and colleagues, 60% of tissues were classed as excluded and 29% as inflamed (4), while in Hammerl and colleagues (8) only 26% were excluded and 46% inflamed. Both articles took different approaches to select and analyze the tissues leading to large contrasts in phenotypes. A comparison of multiple exclusion methods on the same tissue subset would be required to identify the most representative technique. A limitation of these IHC-based TIP analyses is the degree of cell heterogeneity that may be present within the tumor. For example, in some sections, the tumor islands appeared as dense tumor cell colonies, whereas in others the colonies were less dense and showed the presence of fibroblastic cells. However, the analysis does not differentiate between these types of tumor zone organization.

Intratumoral heterogeneity (ITH) has been studied in relation to immune cell phenotype (41); however, little is known on the ITH of the spatial localization of immune cells. Here, we identified multiple phenotypes within a single tissue section that also revealed critical insights on how these phenotypes can vary across tumor tissues. ITH for the number of infiltrating lymphocytes has been shown to impact immunotherapy response (42), and we would add to this observation that variations in TIPs across the tissues may also reflect the efficacy of immunotherapy, and the type of TIP present may also explain the residual disease in areas with a desert or excluded TIP.

VCAN expression and localization was identified to have an association with T-cell location within the TME. VCAN has been shown to be associated with immunity and T-cell trafficking (43) through binding of chemokines (44, 45) and direct interactions with immune cells through CD44 (46) and Toll-like receptors (47–49). Comparisons of VCAN against the type of TIP identified VCAN expression around the epithelium to associate with immune exclusion. Tumor cells have been reported to produce VCAN as part of the pericellular matrices that associated with tumor cell invasion (50) and smooth muscle cell migration (51). Tumor pericellular matrices with high levels of hyaluronan were found to act as barriers to immune cell migration (52), and there may be a similar role for VCAN expression in the epithelial zone of excluded tissues. VCAN levels are further regulated by the action of members of the A Disintegrin-like And Metalloproteinase with Thrombospondin motifs (ADAMTS) family of metalloproteinases (53). Proteolysis by ADAMTS enzymes generates a VCAN fragment called versikine which can lead to an increase in CD8^+^ T-cell infiltration through an increase in immune cell activation and migration (32, 54). In support of this, we found that versikine correlated with stromal VCAN in an inflamed phenotype.

In TNBC, VCAN is expressed by both tumor cells and fibroblasts (25). This was confirmed from our RNAscope analysis. VCAN can be expressed as different isoforms which have been shown to have independent roles. The V0 and V1 isoforms can increase the proliferation of tumor cells (55) while the V2 isoform has the opposing effect (56). The V3 isoform was found to have both protumor and antitumor effects (57), and Kischel and colleagues identified the fifth isoform V4 as a putative breast cancer specific isoform (26). Our RNA analysis identified V0 and V1 isoforms in all tissues, with no isoform or pattern of isoforms to be cell type specific. CAFs had a higher expression of all isoforms in comparison to fibroblasts from noncancerous tissue. Coculturing the mammary fibroblast cell line HMF3S with TNBC cell lines led to an upregulation in VCAN expression but the increase was independent to the cell line showing there is a level of limitation or control. We looked at the major posttranslational modification of VCAN, CS-GAGs. Only inflamed tissues showed a correlation between VCAN and CS. This may be due to other CS proteoglycans being present. BGN and DCN are highly expressed CSPGs in TNBC and predicted to carry two and one CS attachment sites, respectively (58). The expression of both these CSPGs was predominantly stromal and showed no association with phenotype suggesting poor correlations of VCAN and CS in excluded tissues may be due to the length and number of CS chains.

The molecular structure of CS can further vary through sulfation patterns on the disaccharide polymer backbone. Changes in the location of the sulfate group can affect the structural conformation of the CS chains (59), which as previously mentioned can affect the binding capabilities of the isoforms. The CS-A/C ratio was significantly different between the stromal areas of inflamed and excluded tissues. With CS-A having greater binding abilities to chemokines and T cells than CS-C (36), a higher ratio may lead to the attraction of immune cells, whereas a lower ratio, as seen in the stroma of excluded tissues, may limit a chemokine gradient toward the tumor. The effect of the different isoforms was tested through T-cell invasion assays where enrichment for CS-A increased invasion, while enrichment of CS-C reduced invasion. The latter could be overcome by removal of the CS chains using a ChABC treatment. Interestingly, it was the tumor cell line HCC38 that produced a CS-C enriched pattern of CS similar to that identified in excluded tissues, whereas the fibroblast cell line produced a CS-A enriched pattern similar to that identified in inflamed tissues. Our exploration of VCAN and CS isomer role in T-cell trafficking is currently limited to *in vitro* models, and it would be important to also test these observations in an *in vivo* model. This could be done using an injectable orthotopic tumor model, where tumor and fibroblast cell lines with edited CS isomer expression could be correlated with the immune infiltrate.

To conclude, VCAN is highly expressed within tumors and its location is a predictor for defining inflamed or excluded TIPs. The type of CS patterns determines whether VCAN supports or inhibits T-cell trafficking, and we hypothesize that targeting CS-C within the stroma could lead to the increase in T-cell migration toward tumor islands. Chondroitin C lyase could be used to degrade the CS-C isoforms which may then provide an immunologically permissible environment which enables antibody- or cell-based cancer immunotherapies.

## Supplementary Material

Supplementary Figure 1 Supplementary Figure 1 shows how VCAN was enriched and mass spectrometry analysis of the sample

Supplementary Figure 2 Supplementary Figure 2 shows CS analysis from FFPE tissue sections

Supplementary Figure 3 Supplementary Figure 3 describes the transwell model

Supplementary Figure 4 Supplementary Figure 4 shows different method of TIP analysis and the TIPs from different subsets of tissues.

Supplementary Figure 5 Supplementary Figure 5 outlines the analysis of matrix proteins in TMAs.

Supplementary Figure 6 Supplementary Figure 6 shows VCAN analysis in the TIPs from the second tissue subset

Supplementary Figure 7 Supplementary Figure 7 shows representative images of each phenotype

Supplementary Figure 8 Supplementary Figure 8 summarises the results identified.
