# Supplementary figures and images for: Versican Associates with Tumor Immune Phenotype and Limits T-cell Trafficking via Chondroitin Sulfate
Source: Cancer Res Commun. 2024 Apr 3;4(4):970–85. doi: 10.1158/2767-9764.CRC-23-0548 (PMC10989462; doi:10.1158/2767-9764.CRC-23-0548)

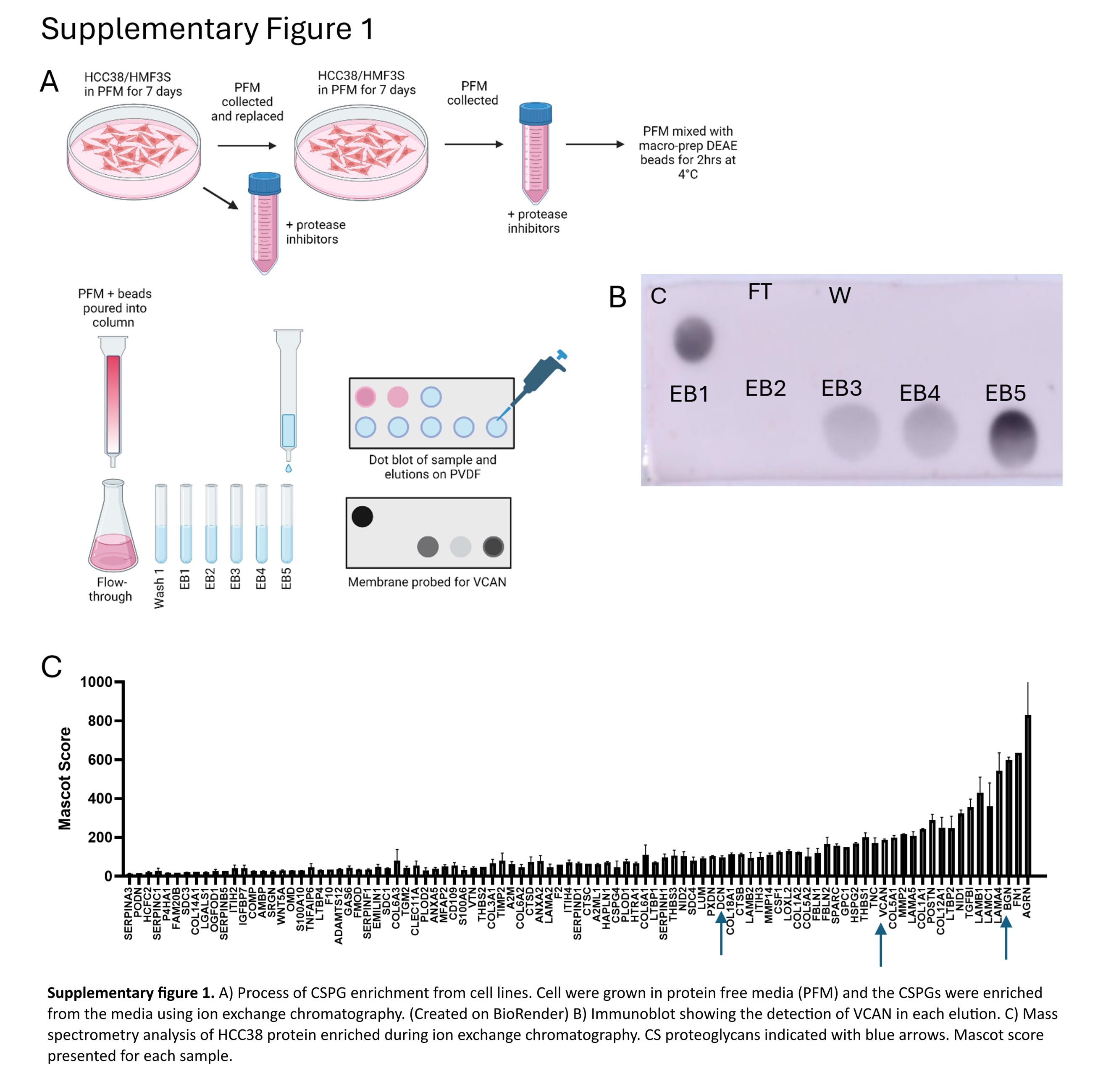

Supplement: Supplementary Figure 1 — shows how VCAN was enriched and mass spectrometry analysis of the sample [file crc-23-0548-s01.jpeg]

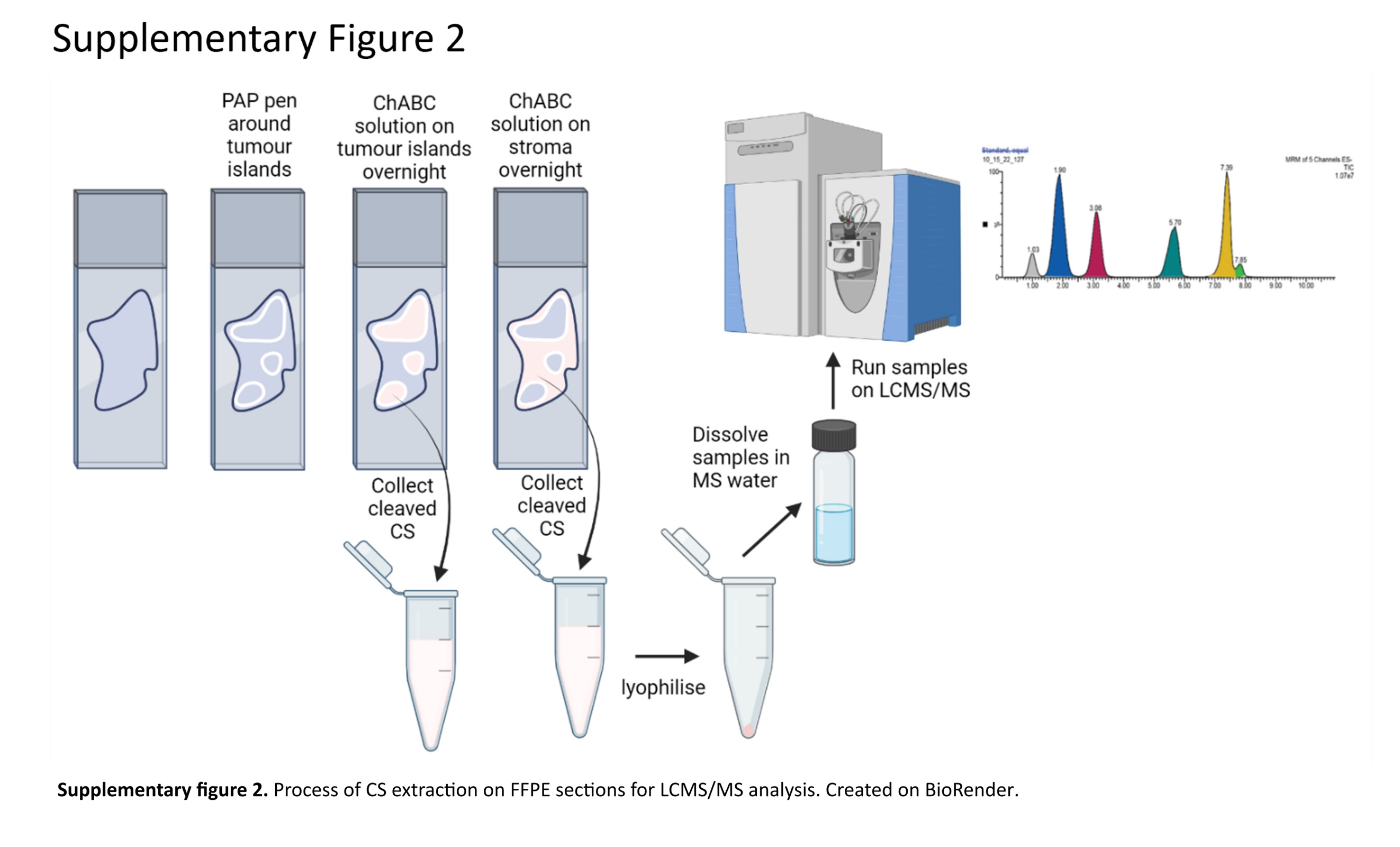

Supplement: Supplementary Figure 2 — shows CS analysis from FFPE tissue sections [file crc-23-0548-s02.jpeg]

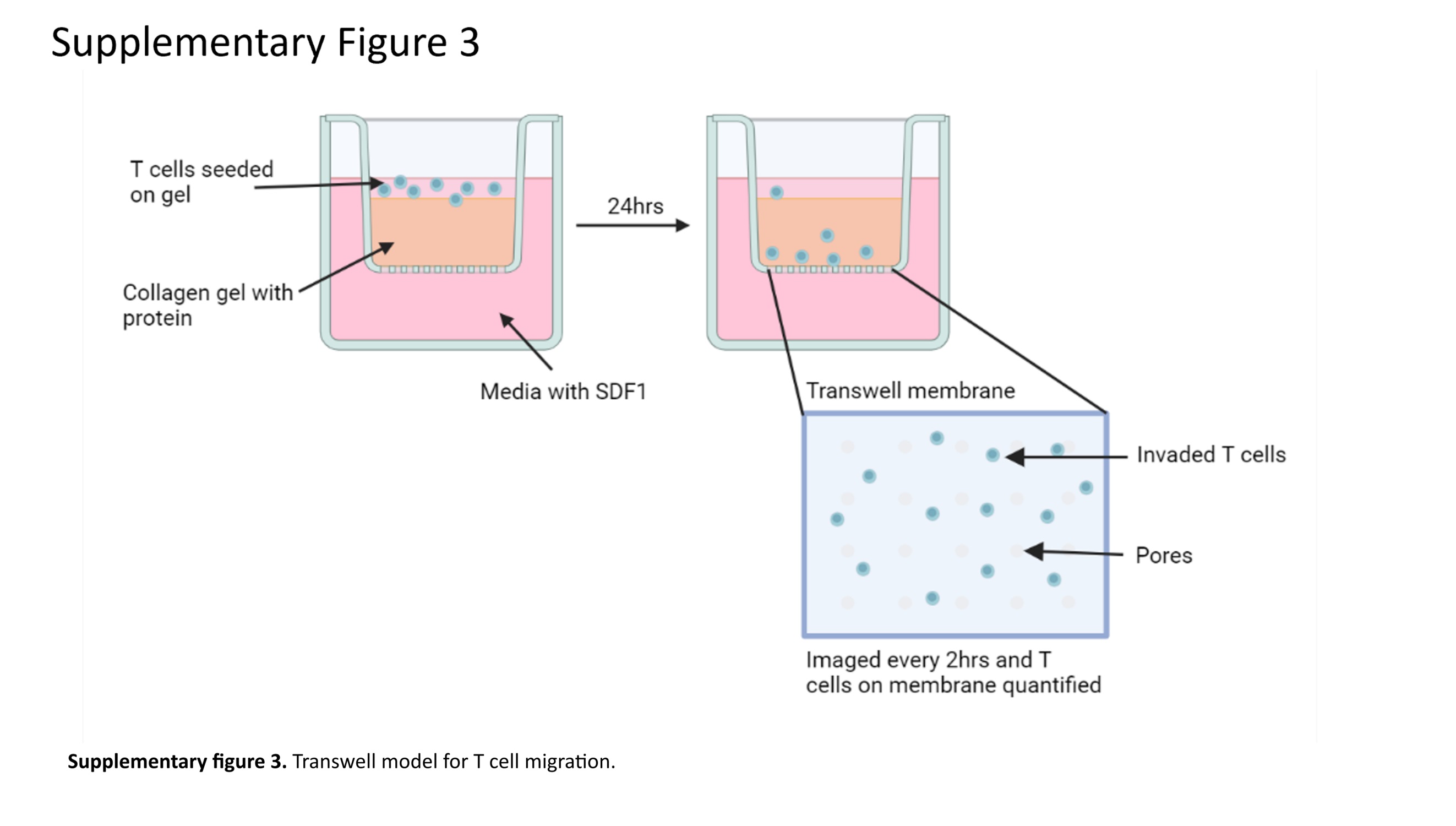

Supplement: Supplementary Figure 3 — describes the transwell model [file crc-23-0548-s03.jpeg]

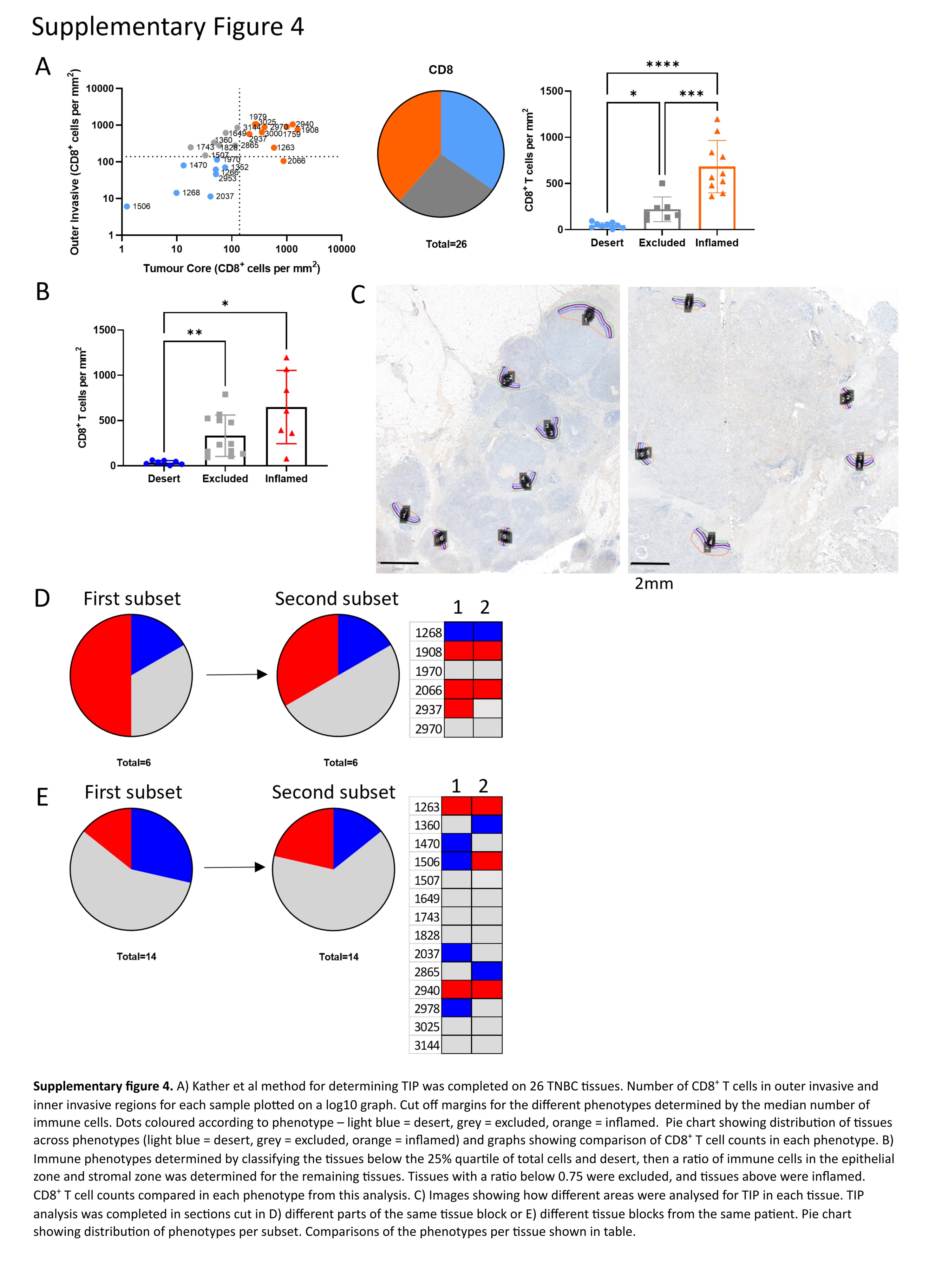

Supplement: Supplementary Figure 4 — shows different method of TIP analysis and the TIPs from different subsets of tissues. [file crc-23-0548-s04.jpeg]

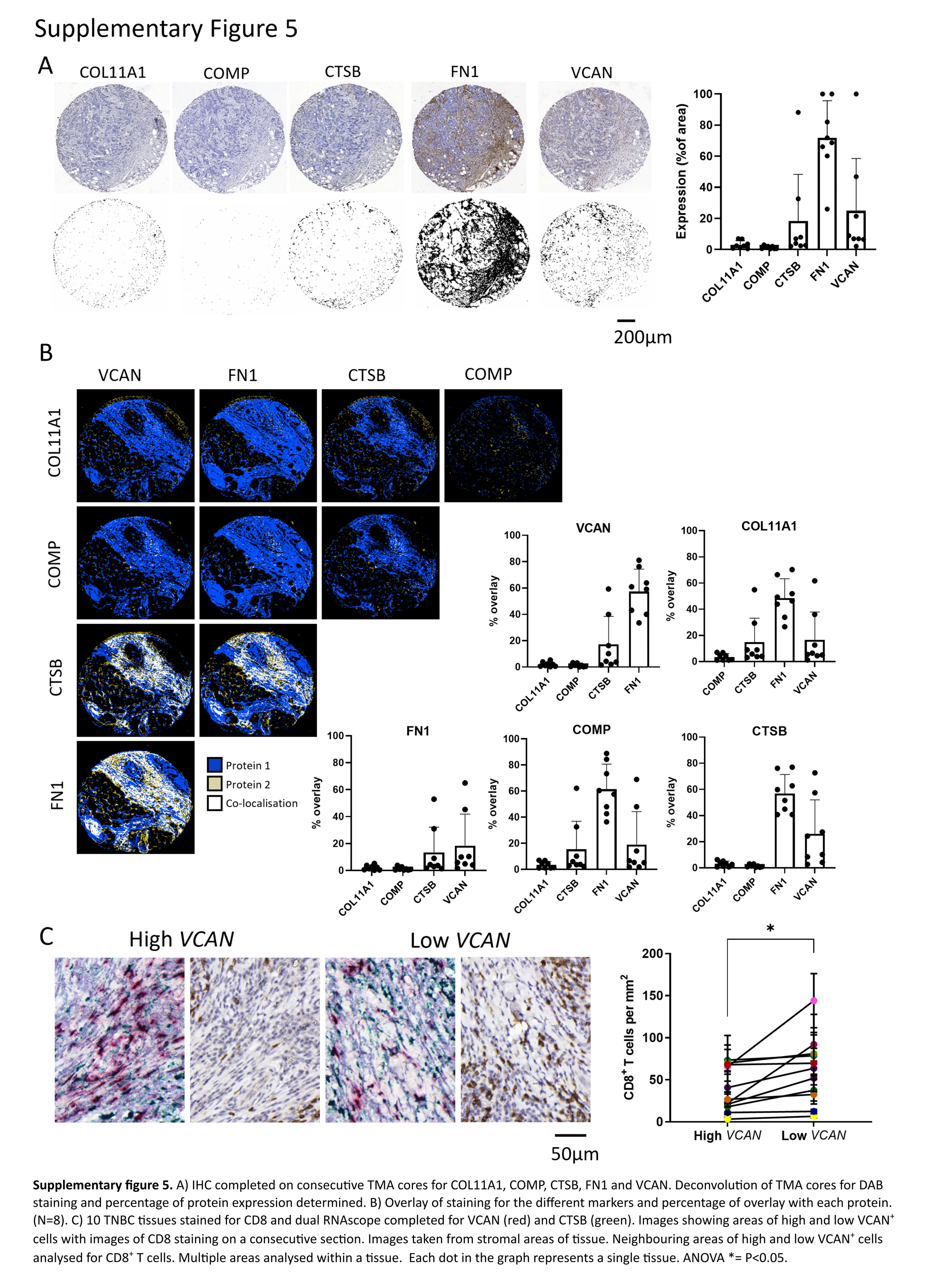

Supplement: Supplementary Figure 5 — outlines the analysis of matrix proteins in TMAs. [file crc-23-0548-s05.jpeg]

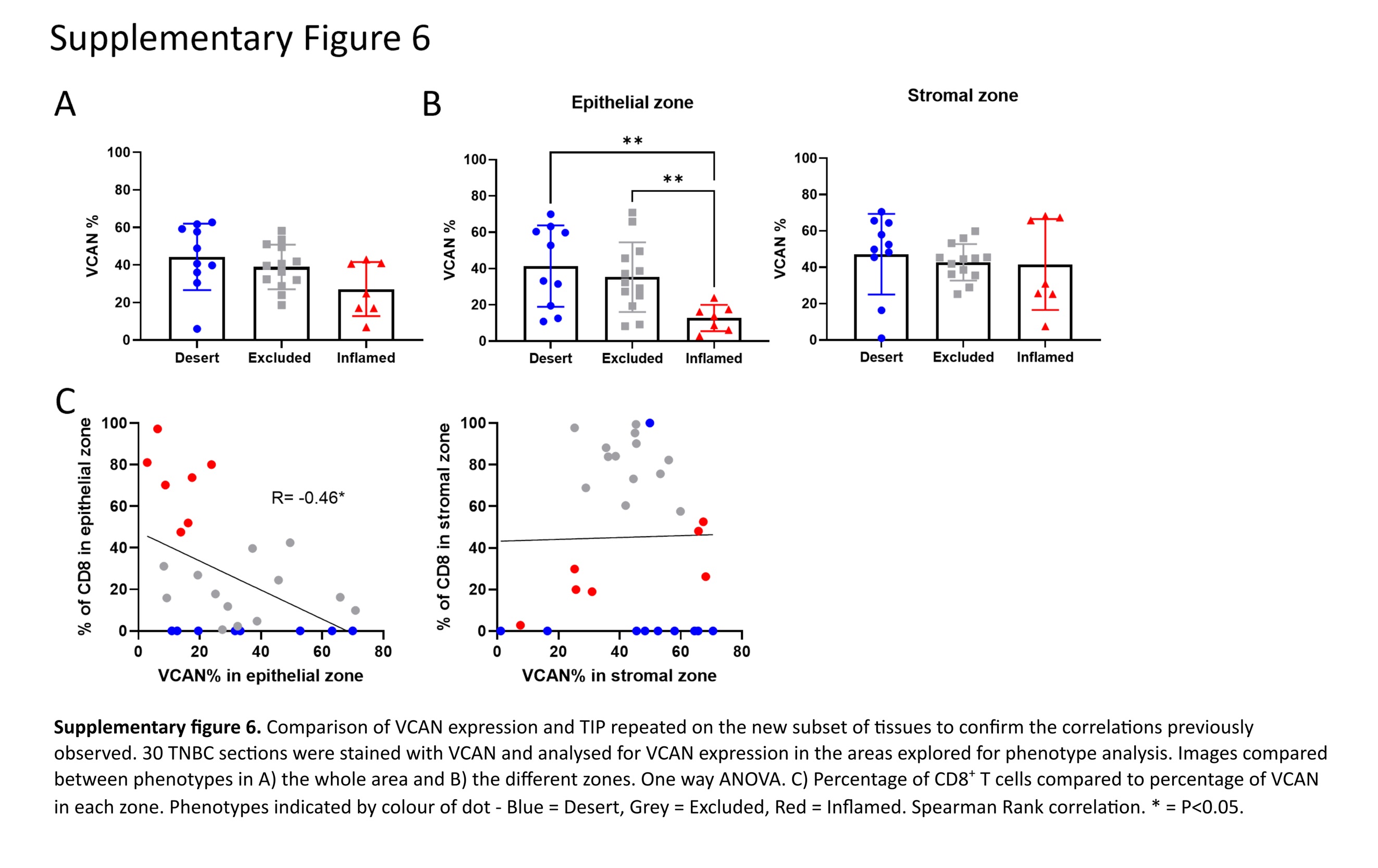

Supplement: Supplementary Figure 6 — shows VCAN analysis in the TIPs from the second tissue subset [file crc-23-0548-s06.jpeg]

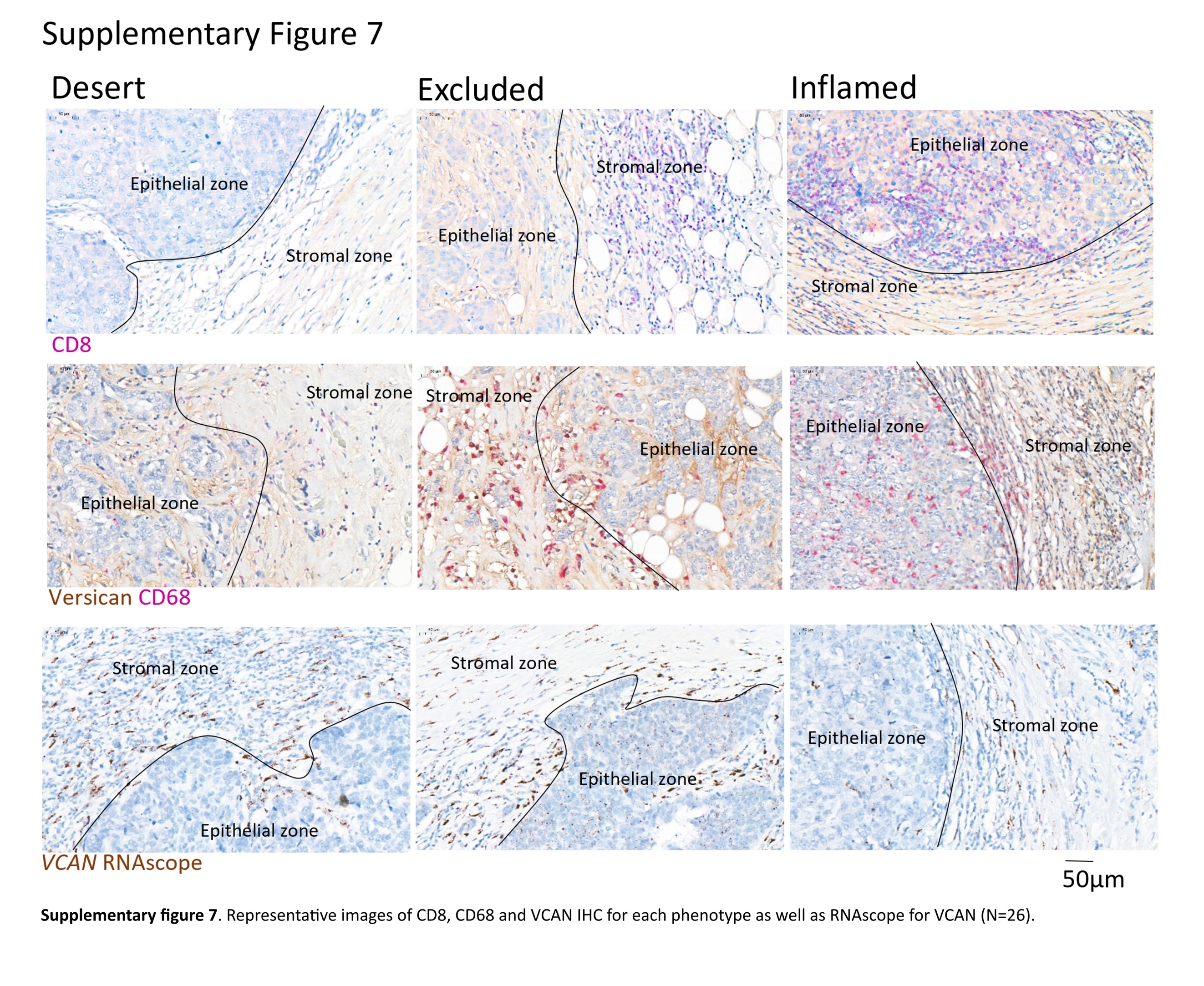

Supplement: Supplementary Figure 7 — shows representative images of each phenotype [file crc-23-0548-s07.jpeg]

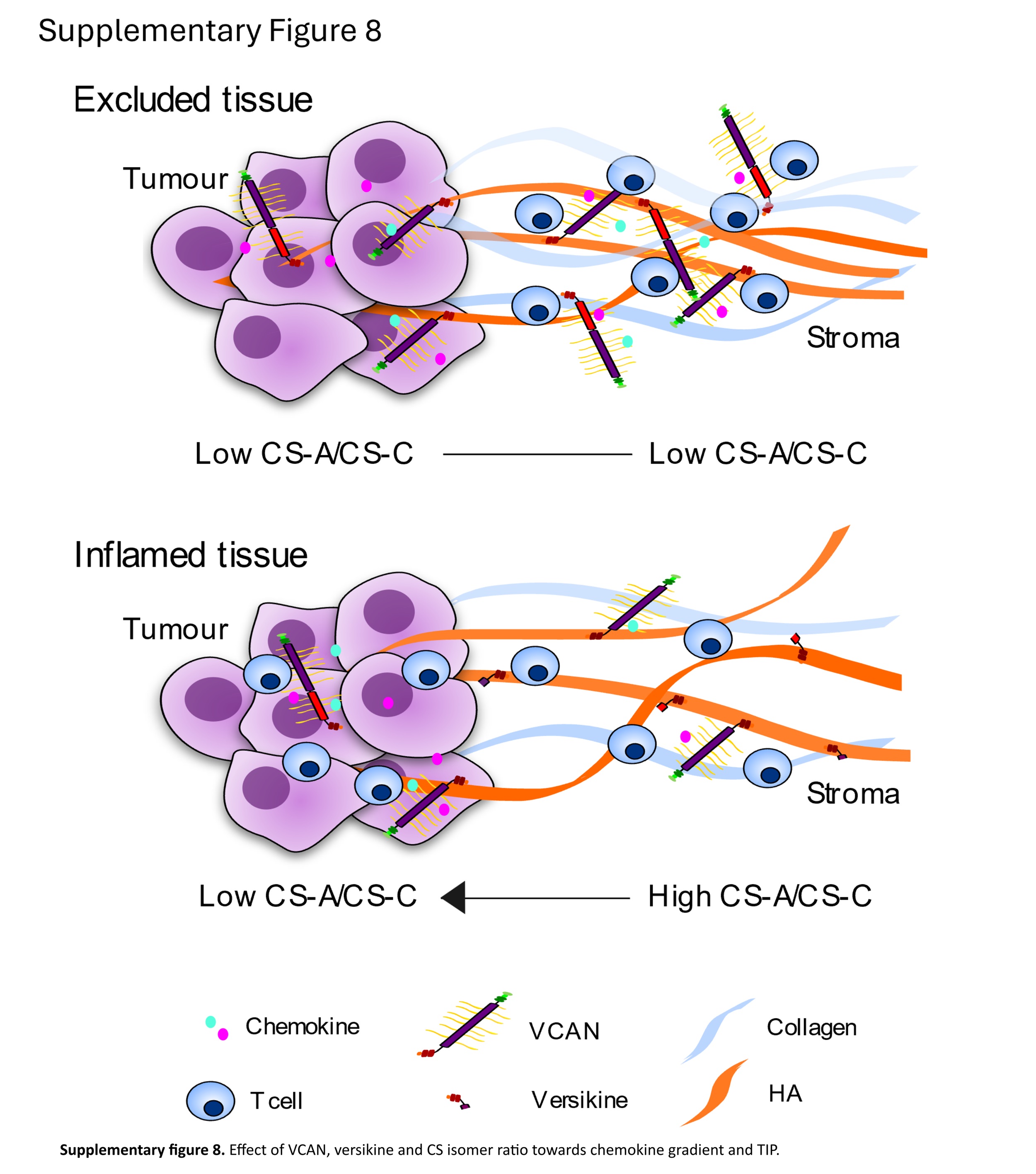

Supplement: Supplementary Figure 8 — summarises the results identified. [file crc-23-0548-s08.jpeg]
